# Non-apoptotic cell death associated with perturbations of macropinocytosis

**DOI:** 10.3389/fphys.2015.00038

**Published:** 2015-02-16

**Authors:** William A. Maltese, Jean H. Overmeyer

**Affiliations:** Department of Biochemistry and Cancer Biology, University of Toledo College of Medicine and Life SciencesToledo, OH, USA

**Keywords:** macropinocytosis, methuosis, vacuoles, non-apoptotic death, necrosis

## Abstract

Although macropinocytosis is widely recognized as a distinct form of fluid-phase endocytosis in antigen-presenting dendritic cells, it also occurs constitutively in many other normal and transformed cell types. Recent studies have established that various genetic or pharmacological manipulations can hyperstimulate macropinocytosis or disrupt normal macropinosome trafficking pathways, leading to accumulation of greatly enlarged cytoplasmic vacuoles. In some cases, this extreme vacuolization is associated with a unique form of non-apoptotic cell death termed “methuosis,” from the Greek *methuo* (to drink to intoxication). It remains unclear whether cell death related to dysfunctional macropinocytosis occurs in normal physiological contexts. However, the finding that some types of cancer cells are particularly vulnerable to this unusual form of cell death has raised the possibility that small molecules capable of altering macropinosome trafficking or function might be useful as therapeutic agents against cancers that are resistant to drugs that work by inducing apoptosis. Herein we review examples of cell death associated with dysfunctional macropinocytosis and summarize what is known about the underlying mechanisms.

## Introduction

Macropinocytosis is a clathrin-independent endocytic process whereby mammalian cells internalize extracellular fluid inside vesicles formed by closure of actin-rich plasma membrane protrusions termed ruffles (Swanson and Watts, 1995; Kerr and Teasdale, 2009). This process is best characterized in dendritic cells and macrophages, where it plays an important role in antigen uptake and processing. However, macropinocytosis also occurs in a broad spectrum of normal and transformed cell types. In cancer cells it appears to contribute to cell growth by helping to maintain nutrient supplies (Commisso et al., 2013; Qian et al., 2014). Historically, insights into the molecular mechanisms that regulate macropinocytosis lagged behind knowledge about receptor-mediated endocytosis. Nevertheless, progress over the past decade has revealed that macropinocytosis can occur either constitutively or in response to external stimuli, such as EGF (Bryant et al., 2007; Swanson, 2008). Initial steps in formation and internalization of macropinosomes depend on cortical actin rearrangements, orchestrated by Rho-family GTPases, and localized changes in membrane phosphoinositide composition (Swanson, 2008; Egami et al., 2014). Once inside the cell, macropinosomes can recycle (Falcone et al., 2006; Bryant et al., 2007; Donaldson et al., 2009) or mature through endosomal intermediates to merge with the lysosomal compartment (Racoosin and Swanson, 1993). GTPases of the Arf and Rab families play key roles in these processes (Sun et al., 2003; Donaldson et al., 2009; Feliciano et al., 2011). Recent studies by our group and others have demonstrated that dysregulation of macropinocytosis, triggered by molecular or pharmacological manipulation of cultured cells, can have striking consequences for cell viability, especially in some types of cancer cells (Overmeyer et al., 2008, 2011; Kitambi et al., 2014). In these instances, hyperstimulation of macropinosome biogenesis, coupled with defects in the pathways for macropinosome recycling and/or maturation, results in extreme cytoplasmic vacuolization. This is followed by a unique form of cytolytic death that is somewhat selective for tumor cells and is distinct from apoptosis or other non-apoptotic cell death pathways (e.g., autosis, paraptosis, necroptosis). The concept that pharmacological induction of this type of cytopathology could be useful in devising new approaches to cancer therapy in tumor cells that are refractory to apoptosis has generated considerable interest (Gilbertson, 2014). In this review we summarize the salient features of several reported cases where perturbations of macropinocytosis have been linked to cell death, and we conclude with a discussion of some of the outstanding questions that need to be resolved as this field of inquiry matures.

## Cell death induced by activation of Ras and Rac pathways

The initial description of cell death triggered by hyperstimulation of macropinocytosis emerged from attempts to characterize an unusual phenotype observed in human glioblastoma cells following ectopic expression of the constitutively active oncoprotein, H-Ras(G12V). Instead of a growth stimulatory effect, Chi et al. (1999) found that Ras expression induced the accumulation of multiple large phase-lucent cytoplasmic vacuoles, followed by caspase-independent cell death. Although this was inferred to be a form of autophagic cell death, the vacuoles did not exhibit the expected double-membrane morphology with degradative contents expected for autophagosomes. In a subsequent study, we established that the Ras-induced vacuoles were in fact derived from macropinosomes, based on electron microscopy demonstrating their origination from plasma membrane projections (Overmeyer et al., 2008). Consistent with the known role of Ras in macropinosome biogenesis (Bar-Sagi and Feramisco, 1986; Li et al., 1997; Porat-Shliom et al., 2008), immuofluorescence studies revealed that epitope-tagged H-Ras(G12V) was localized to the membranes of the vacuoles (Overmeyer et al., 2008). Studies with fluorescent fluid-phase tracers suggested that, unlike normal macropinosomes, the Ras-induced vacuoles did not regurgitate their contents by recycling back to the cell surface. Although the vacuoles acquired some characteristics of late endosomes (LAMP1, Rab7), they did not appear to merge with vesicular compartments labeled with acidotrophic dyes (LysoTracker®) or a cathepsin B substrate (Magic Red RR™) (Overmeyer et al., 2008). These findings supported a model wherein constitutive Ras activation caused extreme endosomal vacuolization not just because of an increase in macropinocytotic activity, but because normal pathways for recycling and eventual lysosomal fusion of macropinosome-derived endosomal vesicles were impaired.

**Figure 1:**
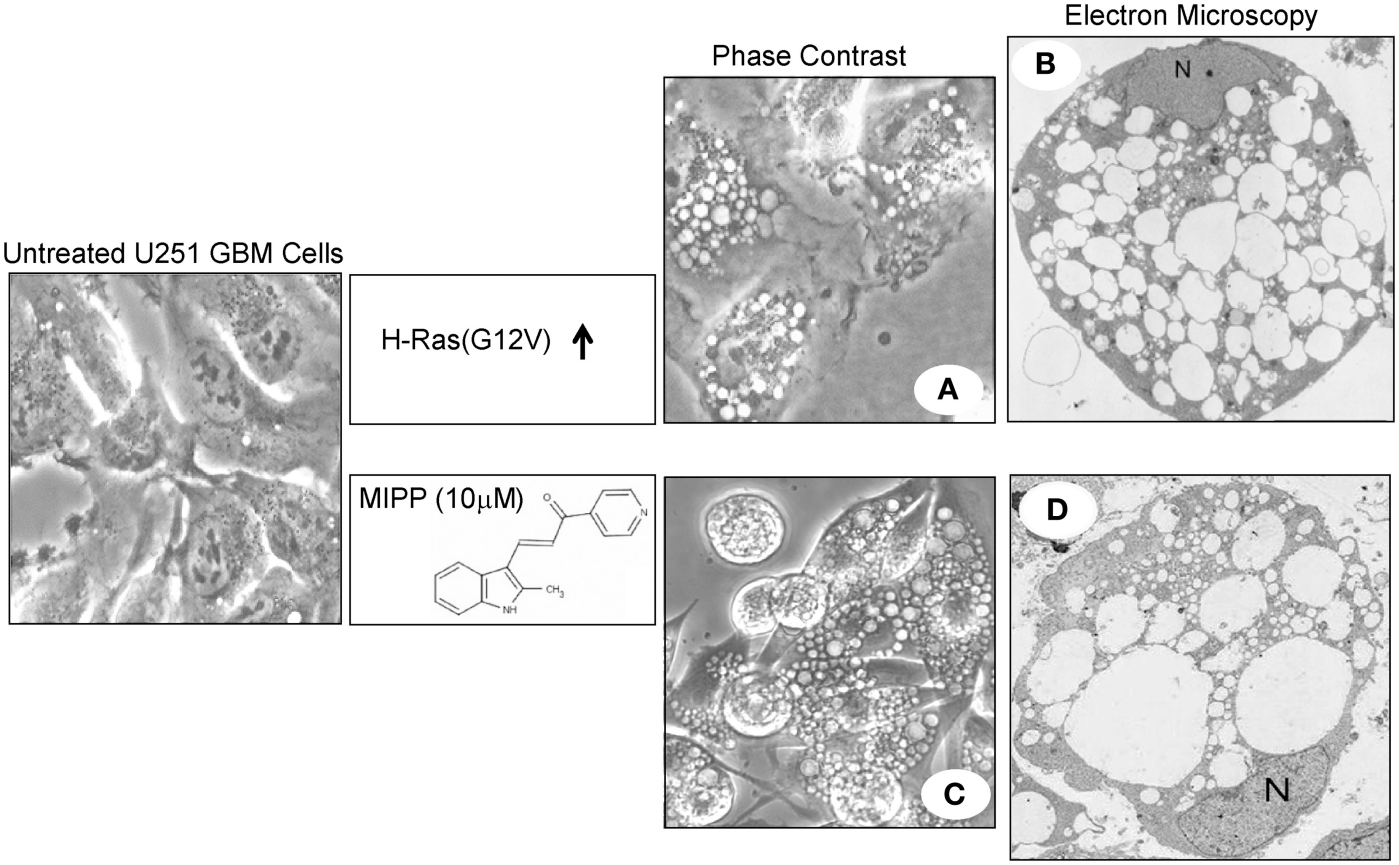
**Examples of extreme vacuolization of macropinosomes associated with non-apoptotic cell death (methuosis) in U251 glioblastoma cells**. **(A,B)** Show phase contrast and electron microscopy images of a stable U251 cell line in which overexpression of H-Ras(G12V) was induced for a period of 4 days (reprinted with permission from Overmeyer et al., 2008). Similar images of U251 cells treated with the indolyl chalcone, MIPP, were obtained after 3 days **(C)** or 2 days **(D)** of drug treatment (reprinted with permission from Overmeyer et al., 2011).

Glioblastoma cells expressing Ras(G12V) lost viability over a period of 4–6 days (Overmeyer et al., 2008). The morphological features of the dying cells were distinct from cells undergoing apoptosis. In particular, the cytoplasmic space was almost entirely occupied by massive clear vacuoles, but the nuclei did not undergo chromatin condensation or fragmentation typically associated with apoptosis (Figure 1). As vacuolization became more extreme, the vacuolated cells exhibited signs of membrane disruption reminiscent of necrosis. Although autophagosomes accumulated in cells expressing activated Ras, these structures were distinct from the macropinosome-derived vacuoles (Overmeyer et al., 2008). Others have shown that Ras-induced autophagy can contribute to non-apoptotic death in some cell types (Elgendy et al., 2011; Wang et al., 2014), but we found that suppressing the expression of autophagy proteins (e.g., Beclin-1) did not prevent Ras-induced vacuolization or cell death in glioblastoma cells (Overmeyer et al., 2008). The foregoing observations, combined with the inability of a broad-spectrum caspase inhibitor (zVAD-fmk) and the necroptosis inhibitor (necrostatin) to protect glioblastoma cells from Ras-induced cell death, prompted us to propose that the degenerative changes associated with dysregulation of macropinocytosis could represent a novel type of necrotic cell death (Overmeyer et al., 2008). Because of the connection to macropinocytosis (often referred to as cell drinking), we coined the term “methuosis” (from the Greek word *methuo*, which means to drink to intoxication) to describe this form of cell death.

Our follow-up studies (Bhanot et al., 2010) provided insights into the mechanisms responsible for Ras-induced methuosis by demonstrating that the Rac1 GTPase is a key downstream mediator. Extreme vacuolization of endosomal compartments induced by H-Ras(G12V) was observed only when Ras expression reached a level sufficient to promote an increase in the activation of endogenous Rac1. Moreover, a specific inhibitor of Rac1 activation (EHT1864) impeded the accumulation of Ras-induced vacuoles. Finally, ectopic expression of activated Rac1, but not other Rho GTPases, was able to mimic the morphological effects of activated H-Ras. The importance of Rac1 as a mediator of Ras-stimulated macropinocytosis was not surprising, in light of previous reports by others establishing the role of Rac1 in the initial steps of macropinosome formation and trafficking (Ridley, 2001; Fujii et al., 2013). However, the difficulty of envisioning a scenario where stimulation of macropinocytosis alone could account for the development of extreme vacuolar cytopathology prompted us to postulate that Arf6, a GTPase known to function in macropinosome recycling (Radhakrishna et al., 1999; Grant and Donaldson, 2009), might be affected by constitutive activation of Ras (Bhanot et al., 2010). We found that in glioma cells expressing H-Ras(G12V), there was an inverse relationship between the activation states of endogenous Rac1 and Arf6. That is, as the relative amount Rac1-GTP increased, the amount of Arf6-GTP decreased. The decline in the pool of active Arf6 was linked to Rac1-mediated activation of an Arf6 GAP, GIT-1, since the decline in active Arf6 was abrogated in cells where GIT-1 expression was suppressed by shRNA. These findings suggested that Ras-induced methuosis might be due to the combined effects of an increase in macropinosome formation and a decrease in macropinosome recycling (Bhanot et al., 2010). However, the apparent inability of the enlarged LAMP1-positive vacuoles to be eliminated by eventual fusion with lysosomes suggests that additional defects at the late endosome-lysosome interface also may contribute to the methuosis phenotype triggered by Ras.

Although Ras-induced vacuolization of macropinosome compartments has been studied mainly in human glioma cells (Overmeyer et al., 2008; Bhanot et al., 2010), similar observations in gastric carcinoma cells (Chi et al., 1999) and osteosarcoma cells (Bhanot et al., 2010), suggest that this novel effect of Ras overexperession is not restricted to brain cancers. In fact, a report showing that expression of activated Ras in *Aspergillus fumigatus* causes excessive vacuolar expansion and lysis of hyphal compartments (Fortwendel et al., 2011) has raised the possibility that a similar Ras-mediated methuosis pathway might be conserved in lower eukaryotes. These surprising findings are at odds with the large body of work showing that endogenous Ras proteins with activating mutations typically promote cell proliferation and tumor progression, rather than cell death (Downward, 2003; Shaw and Cantley, 2006). Indeed, our work with a “tunable” expression system suggests that the hyperstimulation of macropinocytosis and attendant vacuole formation require artificially high levels of ectopic Ras(G12V) expression (Bhanot et al., 2010). Nevertheless, by gaining a better understanding of the mechanisms that trigger this unusual form of cytopathology, it may be possible to develop pharmacological strategies to induce methuosis in a therapeutic context to kill cancer cells that are resistant to apoptosis because of tumor suppressor mutations or heightened DNA repair capacity.

## Methamphetamine-induced perturbations of macropinocytosis

In the course of studies aimed at identifying potential mechanisms whereby methamphetamine (METH) can cause death of neurons in the central nervous system, Nara et al. (2010) observed a unique cell death phenotype reminiscent of methuosis in differentiated cultures of SH-SY5Y neuroblastoma cells. In particular, they noted that when cells were treated with METH, they became filled with phase-lucent vacuoles and began to die by a caspase-independent process within 24 h. They concluded that the vacuoles were derived from macropinosomes, based on their incorporation of high-molecular-weight fluid-phase dextran tracers and prevention of the phenotype by inhibitors of macropinocytosis like cytochalasin D and amiloride. METH-induced perturbation of macropinocytosis was accompanied by accumulation of autophagosomes. However, as in the case of Ras-induced methuosis, the autophagosomes were distinct from the macropinosome-derived vacuoles and autophagy was dispensable for cell death, as evidenced by studies with autophagy inhibitors (Nara et al., 2010).

In a follow-up study, the same investigators provided evidence that hyperstimulation of macropinocytosis in neuroblastoma cells exposed to METH involves activation of Ras and Rac1, based on immunofluorescence localization of activated forms of these GTPases on the METH-induced vacuoles (Nara et al., 2012). Moreover, they showed that both the Rac inhibitor, EHT1864, and the Ras farnesylation inhibitor, farnesyl thiosalicylic acid, could inhibit the formation of vacuoles. In seeking to explain the possible mechanism for the cytotoxic effects of METH, it was noted that proteolytic activation of the lysosomal enzyme, cathepsin L, was impaired in cells treated with METH. Thus, the authors proposed that cell death might be precipitated by defects in lysosomal function. The precise relationship between increased incoming macropinosome traffic and lysosomal dysfunction remains unclear. The model suggested by Nara et al. (2012) postulates that lysosomal defects arise from alkalization due to an abnormally high level of fusion with incoming macropinosomes. The evidence for normal fusion of macropinosomes with lysosomes was the co-localization of some FITC-dextran-labeled vacuoles with LAMP1. However, it is well established that LAMP1 can be detected on non-lysosomal compartments, such as late endosomes and late-stage macropinosomes (Humphries et al., 2011; Egami and Araki, 2012; Pols et al., 2013). Thus, it remains possible that, just as in Ras-induced methuosis, there could be a block in trafficking between macropinosome-derived endosomal vacuoles and lysosomal compartments in cells treated with METH. Such a block in endolysosomal trafficking could account for the impaired proteolytic maturation of cathepsin L, which depends on delivery of procathepsin L to lysosomes from late endosomal vesicles (Ishidoh et al., 1999).

Another explanation for the apparent endolysosomal defects and neuronal toxicity of METH has been suggested by the work of Cubells et al. (1994). In their model, METH can act directly as an acidotrophic weak base to disrupt endosomal and lysosomal pH gradients, promoting both osmotic swelling of these compartments and redistribution of dopamine to generate toxic metabolites. As studies move forward to address the potential involvement of dysfunctional macropinocytosis in the neurotoxicity of METH, it will be important to consider the critical question of whether studies done with cultured cells exposed to millimolar concentrations of METH truly reflect the mechanisms of cytotoxicity that operate in the brains of METH abusers, where physiological levels of METH may be much lower.

## Death by macropinocytosis induced via activation of a receptor tyrosine kinase

Ligand-mediated activation of the EGF receptor and other receptor tyrosine kinases typically results in their incorporation into clathrin-coated endosomes, where signaling can persist until the receptors are internalized into multivesicular bodies and directed to lysosomes for degradation (Katzmann et al., 2002). However, some neurotrophin receptors (e.g., TrkA) appear to be capable of generating prolonged signals because they reside in a distinct population of comparatively stable endosomal vesicles generated through a Rac-dependent macropinocytotic process (Valdez et al., 2007). In contrast to its role in promoting survival and differentiation of normal neuronal cells, activation of TrkA in neuroblastoma or medulloblastoma cells can trigger cell death (Chou et al., 2000; Lavoie et al., 2005; Li et al., 2010). Most interesting in relation to this review, Li et al. (2010) reported that NGF stimulation of Daoy medulloblastoma cells stably expressing TrkA causes hyperstimulation of macropinocytosis, extreme cellular vacuolization and caspase-independent cell death.

As in the preceding examples of cell death associated with abnormal macropinocytosis, siRNA-mediated knockdown of autophagy proteins (e.g., Beclin-1, Atg5, LC3) did not prevent the accumulation of vacuoles or cell death in NGF-stimulated medulloblastoma cells (Li et al., 2010). However, some of the macropinosome-derived vacuoles were capable of incorporating the autophagosome marker, EGFP-LC3, and merging with lysosomes. A possible explanation for these paradoxical observations is suggested by the work of Florey et al. (2011), demonstrating that ectopically expressed GFP-LC3 can be recruited directly to macropinosomes, where it may play a role in downstream fusion with endosomal and lysosomal compartments. In attempting to examine the possible mechanisms underlying the TrkA-mediated induction of cell death, Li et al. (2010) made the intriguing observation that a specific inhibitor of casein kinase 1 (CK1) was able to block both the induction of macropinocytosis and the associated death of the medulloblastoma cells caused by activation of TrkA. At present it remains unclear which targets of CK1 might be involved in regulating macropinocytosis. Nevertheless, this finding is important because it supports the concept that macropinocytosis is mechanistically linked to cell death in this system.

## Macropinosome vacuolization and cell death induced by small molecules

Following our initial description of Ras-induced cell death associated with vacuolization of macropinosome-derived endosomal compartments (methuosis), we identified a series of small molecules that induced a very similar form of cell death in a Ras-independent manner (Overmeyer et al., 2011; Robinson et al., 2012). The prototype was a synthetic indole-based chalcone termed MIPP; an acronym for 3-(2-methyl-1H indol-3-yl)-1-(4-pyridinyl)-2-propen-1-one. At low micromolar concentrations MIPP triggered rapid accumulation of numerous phase-lucent cytoplasmic vacuoles when applied to human glioblastoma cells (Figure 1). Time-lapse microscopy revealed waves of macropinosomes entering the cells between 13 and 80 min after addition of the compound (Overmeyer et al., 2011). Nascent vesicles rapidly coalesced to form large vacuoles, and eventually the influx of macropinosomes slowed down. Treatment of cells with filipin (a cholesterol binding agent) blocked vacuole formation, consistent with the known dependence of macropinocytosis on cholesterol-rich membrane domains (Castro-Obregon et al., 2004). The MIPP-induced vacuoles acquired Rab7 and LAMP1, indicative of a transition from macropinosomes to enlarged late endosomal structures. However, there was little or no overlap between the phase-lucent vacuoles and compartments labeled with markers for lysosomes (LysoTracker, Magic Red RR) or autophagosomes (LC3II), suggesting a defect at the late endosome-lysosome boundary.

Within 2 days after addition of MIPP there was a decline in cellular ATP levels and cell viability (Overmeyer et al., 2011). A non-apoptotic mode of death was supported by the fact that while the plasma membrane was disrupted, the nuclear membrane stayed intact and chromatin remained diffuse (Figure 1). Although there was some caspase activation (PARP cleavage), the caspase inhibitor, zVAD-fmk, did not prevent loss of viability. Overall, except for an accelerated evolution of macropinocytotic vacuoles and a more rapid pattern of cell death, the cytopathology of cells exposed to MIPP was very similar to the methuosis phenotype we described in cells expressing H-Ras(G12V) (Figure 1). It is worth noting that the activation states of Rac1 and Arf6 were not altered by MIPP. However, there was marked decline in active Rab5 (Overmeyer et al., 2011), a GTPase that plays a key role in early endosomal trafficking, including macropinosome maturation (Feliciano et al., 2011). These observations suggest that MIPP operates at stages of macropinosome biogenesis and endolysosomal trafficking downstream from the early steps regulated by Ras, Rac1, and Arf6.

Our continuing studies with synthetic libraries of MIPP-related compounds have identified more potent derivatives (e.g., a 5-methoxy derivative, MOMIPP) (Robinson et al., 2012; Trabbic et al., 2014). The abrogation of methuosis by very minor changes in the structures of these compounds (e.g., changing the configuration of the pyridinyl nitrogen from *para* to *meta*) suggests that the effects of these compounds are due to interactions with specific intracellular protein targets, rather than general covalent protein modification by Michael addition. However, the identities of the targets have not yet been established.

Kitambi et al. (2014) recently reported the results of a phenotypic screen in which they identified an entirely different class of small molecules termed Vacquinols, which have the ability to kill glioblastoma cells through a process involving massive membrane ruffling, accumulation of macropinosome-derived vacuoles, decreased ATP, and rupture of the cell membrane. This process, which they termed catastrophic vacuolization, is insensitive to inhibitors of caspase activation, autophagy and necroptosis, and exhibits most of the hallmarks of methuosis. The lead compound, Vacquinol-1, had excellent pharmacokinetic properties and showed good anti-tumor efficacy against glioblastoma in zebrafish and mouse models. Unlike the indolyl chalcones, the cytotoxic activity of the Vacquinols so far appears to be relatively selective for glioblastoma. Although the protein targets of the Vacquinols remain to be defined, an unbiased shRNA screen revealed that the activity of a MAP kinase kinase, MKK4, was required for induction of macropinocytosis and cell death by Vacquinol-1 (Kitambi et al., 2014). There is some evidence that MKK4 functions downstream of Rac1 in the JNK pathway (Wang et al., 2014), but it remains to be determined how MKK4 might function in macropinosome biogenesis and trafficking.

## Other stimuli causing abnormalities in macropinocytosis

With increasing awareness that hyperstimulation of macropinocytosis or disruption of normal macropinosome trafficking can result in loss of cell viability, it is likely that additional triggers for such forms of cell death will be discovered. Two recent examples illustrate this trend.

The first example comes from studies seeking to identify dysregulated miRNAs in papillary thyroid carcinoma, which revealed miR-199a-3p as a potential tumor suppressor (Minna et al., 2014). Restoration of miR-199a-3p in cells that underexpress this molecule caused the tumor cells to die by a non-apoptotic pathway resembling methuosis, with characteristic accumulation of cytoplasmic vacuoles derived from macropinosomes. Although the precise molecular mechanisms linking miR-199a-3p to this lethal phenotype remain to be defined, the authors noted that a number of genes involved in the regulation of macropinocytosis are among the predicted targets of miR199a-3p.

A second example comes from studies of AS1411, a 26-base G-rich oligonucleotide that binds to nucleolin as an aptamer and selectively induces cell death in a broad spectrum of cancer cells, but not normal cells (Bates et al., 2009; Choi et al., 2010). Reyes-Reyes et al. (2010) reported that after its initial uptake via basal macropinocytosis, AS1411 acts in a nucleolin-dependent manner to further stimulate macropinocytosis in cancer cells. Nucleolin exhibits oncogenic synergism with active Ras and ErbB and co-localizes with these proteins at the plasma membrane (Schokoroy et al., 2013). However, it remains unclear whether the effects of AS1411 on macropinocytosis may be mediated through nucleolin interaction with Ras or its downstream effectors. In addition, it will be important to ascertain if stimulation of macropinocytosis by AS1411 is directly responsible for the selective cytotoxic activity of this aptamer in cancer cells.

## Future directions

**Figure 2:**
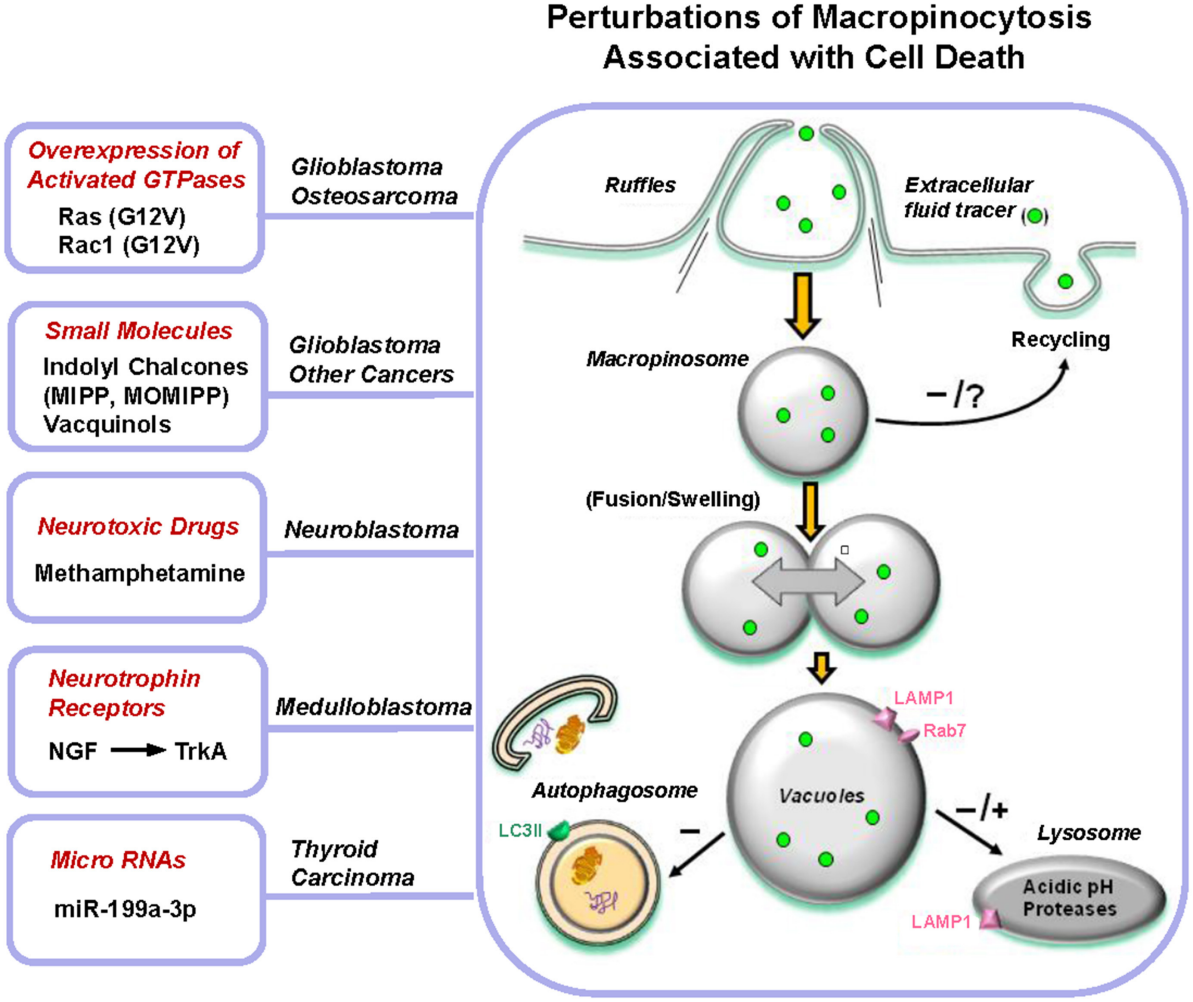
**Perturbations of macropinocytosis associated with cell death**. A molecular or pharmacological stimulus triggers a vigorous increase in membrane ruffling and formation of macropinosomes. This may be sustained (as in Ras or TrkA stimulation) or transient (MIPP treatment). Once generated, the incoming macropinosomes give rise to a multitude of large vacuoles that fill much of the cytoplasmic space. Studies with MIPP indicate that vacuole enlargement occurs through fusion events, although osmotic swelling has not been ruled out conclusively in most cases. Defective recycling of macropinosomes contributes to vacuole formation in specific instances (Ras or Rac overexpression, treatment with MIPP), but this has not been examined in all examples. The vacuoles are able to mature to acquire late endosomal characteristics, such as LAMP1 or Rab7, but they generally remain separate from autophagosomes and are not dissipated by suppressing the expression of autophagy proteins. Conclusions about the ability of the vacuoles to merge with lysosomes vary. The vacuoles generated by Ras, Rac, MIPP, and MiR-199a-3p appear to be defective in their ability to fuse with lysosomes, whereas those generated by TrkA stimulation and METH exhibit some overlap with lysosomal compartments. Ultimately, metabolic failure and loss of membrane integrity occur, but the specific mechanisms linking vacuolization to these sequelae have not been defined.

**Figure 3:**
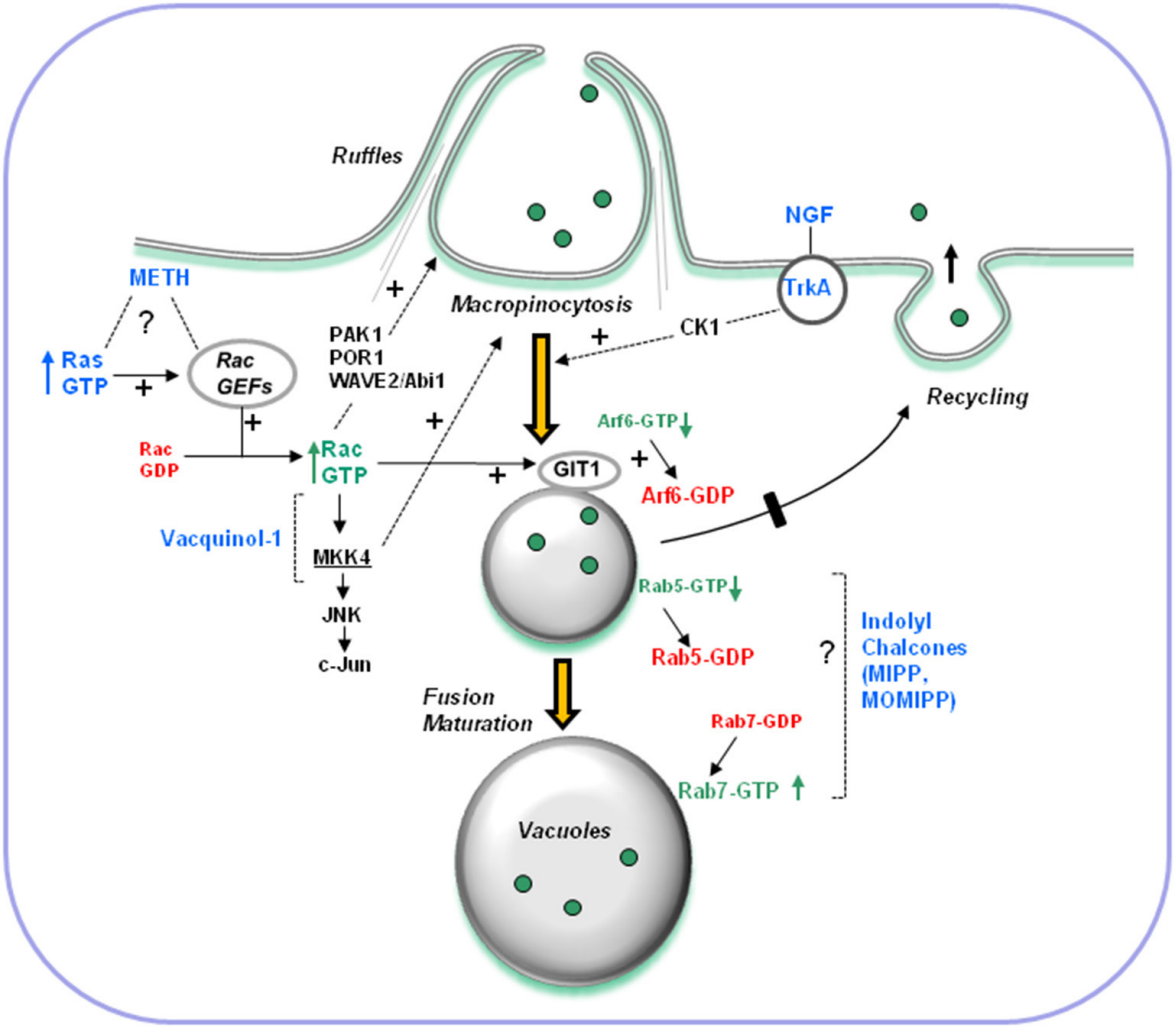
**Overview of signaling pathways implicated in hyperstimulation of macropinocytosis and vacuolization of macropinosome-derived compartments**. Known stimuli discussed in this review are shown in blue. Dashed lines denote pathways where specific mechanisms and intermediates are not yet defined. Some pathways may be operational only in certain cell types. The effects of constitutively active Ras and high concentrations of METH require activation of Rac1. In addition to altering the amount of active Arf6 via GIT-1, sustained activation of Rac1 has the potential to affect multiple protein targets known to function in actin remodeling, membrane ruffling and initial steps of macropinocytosis (e.g., PAK1, POR1, WAVE2). The effects of NGF in cells overexpressing TrkA depend on casein kinase 1 (CK1), but the specific function of CK1 in macropinocytosis is obscure. The effects of Vacquinol-1 require MKK4, a downstream component of the Rac1-JNK stress signaling pathway. It is not yet clear how MKK4 functions in macropinosome biogenesis or trafficking. The compounds MIPP and MOMIPP, have inverse effects on the activation states of Rab5 (macropinosomes and early endosomes) and Rab7 (late endosomes). They probably exert their effects by altering vesicular trafficking steps downstream from those regulated by Rac1 and Arf6.

Although it is well established that macropinocytosis plays a vital role in many different types of cells under normal physiological circumstances, the recognition that artificial perturbation of macropinocytosis can lead to a novel form of non-apoptotic death in cancer cells is a relatively recent development. Figure 2 summarizes the various molecular or pharmacological stimuli that have been reported to cause vacuolization of macropinosome-derived compartments. Common features in all of these scenarios include transient or sustained stimulation of macropinocytosis, dysfunctional recycling and/or lysosomal-directed trafficking of the resulting macropinosomes, and swelling/coalescence of intermediate vesicular compartments. Typically these effects have been documented in cancer cells, where constitutive macropinocytosis can be more active than is commonly appreciated. This has raised the possibility that manipulation of macropinocytosis pathways could represent a novel strategy for killing malignant cells that are inherently resistant to apoptosis, due to mutations in tumor suppressor genes that control responses to DNA damage or oxidative stress (Delbridge et al., 2012). Furthermore, one might envision that inducing cytolysis via accumulation of macropinosome-derived vacuoles could circumvent the well-known ability of tumor cells to adapt to conventional chemotherapeutic drugs by increasing their DNA repair capacity or drug efflux pathways (Bocangel et al., 2002; Dean et al., 2005; Abdullah and Chow, 2013). Our preliminary studies have provided some support for the latter concept by demonstrating that methuosis-inducing compounds, MIPP and MOMIPP, can be effective in killing temozolomide-resistant glioblastoma cells and doxorubicin-resistant breast cancer cells *in vitro* (Overmeyer et al., 2011; Robinson et al., 2012). To further explore the possibility that apoptosis-resistant cancer cells might be vulnerable to an alternative form of cell death triggered by dysfunctional macropinocytosis, it will be important to address several key questions:

1. *Are tumor cells more vulnerable to disruption of macropinocytosis than normal cells and, if so, what is the basis for this difference?* Vacquinol-1 exhibits toxic activity against glioblastoma, but not human fibroblasts or normal embryonic stem cells (Kitambi et al., 2014). MIPP and MOMIPP show variable levels of cytotoxicity against tumor cells in addition to glioblastoma. Yet, as in the case of Vacquinol-1, normal fibroblasts or mammary epithelial cells are less sensitive to these compounds (Overmeyer et al., 2011; Robinson et al., 2012). Interestingly, despite reduced effects on cell viability, treatment of normal fibroblasts or epithelial cells with MOMIPP still causes striking vacuolization of endosomal compartments in these cells (Robinson et al., 2012). Why then does vacuolization have a more severe impact on viability in tumor cells compared with normal cells? One possible explanation could be that the consequences of dysfunctional macropinocytosis and cytoplasmic vacuolization are more immediate in rapidly proliferating cancer cells compared with slower-growing contact-inhibited normal cells. Alternatively, it is possible that cancer cells may be more dependent on macropinocytosis than normal cells for acquisition of nutrients, so that disruption of macropinosome trafficking to the lysosomes has a greater impact on cellular metabolism and ATP levels. Finally, it is conceivable that the vacuoles generated by methuosis-inducing compounds in tumor cells are qualitatively different from those induced in normal cells, either in terms of their origin or their ability to trigger stress pathways that can lead to cell death.
2. *What specific signaling pathways are involved in triggering vacuolization of macropinosome compartments?* The limited information concerning signaling pathways implicated in the hyperstimulation of macropinocytosis or vacuolization of macropinosome-derived compartments is summarized in Figure 3. Activation of Rac1 plays an important role in some cases, particularly those triggered by overexpression of Ras(G12V) or treatment with METH. In other examples, kinases not previously linked to macropinocytosis appear to be involved (e.g., MKK4 in the case of Vacquinol-1, and CK1 in the case of NGF-TrkA). The specific GEF (guanine nucleotide exchange factor) that links Ras to Rac1 in the context of methuosis remains a mystery. Our experiments utilizing shRNAs to suppress the expression of two likely candidates, Tiam1 and Eps8 (a component of the Sos1/Eps8/E3b1 complex), did not reveal any inhibition of H-Ras-induced macropinocytosis and vacuolization (Bhanot et al., 2010).
  As noted earlier, overexpression of Rac1(G12V) can induce the methuosis phenotype directly, independent of Ras. This requires interaction of Rac1 with specific effectors, since neither Cdc42(G12V) nor RhoA(G14V) produced the same phenotype (Kaul et al., 2007). Other Rho-family GTPases have not yet been examined, but RhoG merits consideration since it has been reported to stimulate membrane ruffling and macropinocyctosis (Ellerbroek et al., 2004) and can activate Rac1 in response to EGF or HGF in glioblastoma cells (Kwiatkowska et al., 2012). Identification of the Rac1 effectors involved in promoting methuosis is still incomplete. When expressed at high levels, Rac1(G12V) appears to cause a decline in the level of active Arf6, presumably through stimulation of the Arf6 GAP, GIT-1. While this may account for the block in macropinosome recycling, the basis for hyperstimulation of macropinosome biogenesis remains unclear. One Rac1 effector that could be involved is p21-activated kinase-1 (PAK1), which is known to enhance macropinocytosis by promoting actin remodeling and membrane ruffling (Dharmawardhane et al., 2000). However, in unpublished studies we have noted that co-expression of the PAK1 autoinhibitory domain with Rac1(G12V) does not abrogate macropinosome vacuolization. Other Rac1-reponsive proteins that could play roles in formation of membrane ruffles and macropinosomes include POR1 (Van Aelst et al., 1996) and the WAVE2/Abi1 complex (Innocenti et al., 2004; Dubielecka et al., 2010). Interestingly, Abi1 is one of the predicted targets of miR-1991-3p, which induces macropinocytotic vacuoles and causes non-apoptotic death in papillary thyroid carcinoma cells (Minna et al., 2014).
  With the identification of small molecules, exemplified by MIPP, MOMIPP, and Vacqinol-1, which may have therapeutic potential to induce methuosis-like cell death in cancer, future efforts undoubtedly will focus on identifying the targets of these compounds. Although such studies are in their early stages, we have noted that induction of methuosis by MIPP is distinct from Ras-induced methuosis insofar as it does not entail alterations in the activation states of Rac1 or Arf6. On the other hand, MIPP causes a marked decline in active Rab5 (Overmeyer et al., 2011), which is critical for macropinosome stabilization (Feliciano et al., 2011). This could occur through a number of possible mechanisms, including direct interference of the compound with Rab5 activation by GEFs (e.g., Rabex-5, Rin1), accelerated Rab5 inactivation by GAPs, indirect disruption of the Rab5 cycle due to changes in endosomal membrane structure or pH, or premature recruitment of Rab7, which inhibits Rabex-5 (Rink et al., 2005; Poteryaev et al., 2010; Feliciano et al., 2011). Consistent with the latter idea, the membranes of most of the vacuoles induced by MIPP contained Rab7, and the overall amount of active Rab7 was elevated in MIPP-treated cells (Overmeyer et al., 2011). An important question for future consideration is why these macropinosome-derived vacuoles fail to dissipate by merging with lysosomal compartments, despite showing some signs of late endosomal maturation (i.e., acquisition of Rab7 and LAMP1). At the present time there is no available information about the potential interaction of methuosis-inducing compounds with proteins that maintain endosomal proton gradients or function in late endosome lysosome trafficking pathways.
3. *What is the relationship between vacuolization of macropinosome-derived compartments and cell death?* All of the examples of cell death associated with perturbations of macropinocytosis share some common features, which are summarized in the model depicted in Figure 2. Once vacuolization of macropinosome-derived compartments occurs, the exact execution steps that lead to the demise of the cell remain poorly defined. Depending on the specific stimulus, caspase activation may or may not occur, but it is clear that caspase inhibitors afford little or no protection from death. Moreover, nuclear changes typical of apoptosis are uniformly absent. On the other hand, decreased ATP and loss of membrane integrity, reminiscent of necrosis, appear to be common outcomes in these forms of cell death. A key question is whether vacuolization of endosomal compartments is directly responsible for triggering cell death. Pharmacological approaches to block macropinocytosis by interfering with the actin cytoskeleton (cytochalasin D), Na+/H+ exchange (amiloride) or the vacuolar-type H+-ATPase (bafilomycin-A1) have limited utility for delineating the pathways that lead to cell death, because these agents are themselves cytotoxic when applied to cells for prolonged periods of time. However, studies involving inhibition of specific protein kinases support the notion that there is a causal relationship between dysfunctional macropinocytosis and cell death. For instance, blocking TrkA-induced formation of macropinocytotic vacuoles by treatment of medulloblastoma cells with the CK1 inhibitor, D4476, prevented cell death (Li et al., 2010). Similarly, suppressing the expression of MKK4 in glioblastoma cells impeded the induction of vacuolization by Vacquinol-1, and concomitantly reduced the cytotoxic effect of the compound (Kitambi et al., 2014). A final line of correlative evidence, based on structure-activity relationships of MIPP-related indolyl chalcones, lends credence to the idea that vacuolization of macropinosomes is an important component of the cell death program. Specifically, we noted that minor structural changes that eliminated the ability of compounds to induce macropinosome vacuolization also eliminated their ability to kill glioblastoma cells (Robinson et al., 2012). Notwithstanding this body of evidence, our most recent results suggest that some caution is warranted in concluding that vacuolization of macropinosome-derived compartments is, by itself, sufficient to trigger cell death. In particular, we identified a sub-group of indolyl chalcones that induced robust vacuolization in glioblastoma cells without substantially inhibiting proliferation or viability (Trabbic et al., 2014). This raises the possibility that, in at least some cases, cell death associated with accumulation of macropinosome-derived vacuoles may entail unrecognized pleiotropic effects on vital cellular processes beyond macropinocytosis. In this situation, one might view hyperstimulation of macropinocytosis or disruption of macropinosome trafficking as essential priming events that combine with other cellular insults to cause eventual metabolic collapse. In this regard it will be interesting to study in greater detail the interrelationships between macropinocytotic dysfunction and cellular bioenergetic pathways, stress responses, cytoskeletal architecture and lysosomal integrity.

Even if agents that hyperstimulate macropinocytosis or interfere with macropinosome recycling pathways do not always lead directly to cell death, one might speculate that such agents could have benefits in other contexts. One possibility is that stimulation of macropinocytosis might be utilized to increase fluid-phase drug uptake in resistant cancer cells where induction of ABC transporters promotes rapid efflux of drugs entering via conventional transmembrane routes. Alternatively, one might envision that stimulation of macropinocytosis could be harnessed to facilitate uptake of drugs packaged in nanoparticle delivery vehicles. Finally, recent studies have established that cancer cells utilize Arf6-dependent mechanisms to generate and release plasma-membrane derived microvesicles containing proteases and other factors that can promote invasion and tumor progression (Muralidharan-Chari et al., 2009, 2012). It would be interesting to determine whether molecular or pharmacological manipulations that impede macropinosome recycling might also affect the release of such microvesicles.

### Conflict of interest statement

The authors declare that the research was conducted in the absence of any commercial or financial relationships that could be construed as a potential conflict of interest.
